# Fellow-Workers and the Roll of Honour

**Published:** 1915-01-09

**Authors:** 


### ROUND THE WORLD.

[The Editor Invites Early Information and Contributions Marked "Fellow-Workers."]

### Fellow-Workers and the Roll of Honour.

The Asylum Service has lost a capable officer by the death in action of the late Mr. Sydney Nelson Crowther, who left his post of senior A.M.O. at Surrey County Asylum, Netherne, to enlist on the outbreak of war. The late Mr. Crowther was an old Westminster Hospital man, and after qualifying L.R.C.P., M.R.C.S. in 1903 held the posts of house physician and house surgeon there. As a student he gained experience of field service in the South African War, where he served as a dresser in the Imperial British Yeomanry Hospital. A keen sportsman, he had played both for the United Hospitals Rugby Team and for the British XV. that went round the world some time since. On joining the Army, Mr. Crowther was given a responsible post as a motor-cyclist dispatch rider, and met his death during fulfilment of his perilous duties.

The London Hospital has lost a distinguished student by the death of the late Lieutenant George Henry Chisnall, who had joined the R.A.M.C. in the early days of the war and became attached to the 1st Battalion Cameron Highlanders. An M.B., B.S. Lond. and F.R.C.S. Eng., this brilliant young surgeon, who had held numerous resident appointments at his hospital and won the attention of his teachers by his keen industry, had recently been awarded the Jonathan Hutchinson Prize for an able essay on "Fractures of the Upper Part of the Humerus and their Treatment."

A popular Middlesex Hospital man, the late Lieutenant Henry John Sladen Shields, R.A.M.C., was killed recently. Following his preliminary medical studies at Cambridge, where he distinguished himself on the river, the late Mr. H. J. S. Shields qualified L.R.C.P. Lond., M.R.C.S. Eng. from the "Middlesex" two years ago, and became a graduate of his university last year. He was one of the first medical officers to leave for the Front with the Expeditionary Force, and was attached to the Royal Irish Guards. He escaped the perils of the fierce fighting round Mons, although for some time he was cut off from his regiment, only to lose his life a little later.

The late Major Edwin Bedford Steel, R.A.M.C., who died at La Clytte of wounds received in action, was educated at Trinity College, Dublin, and qualified M.B., B.Ch. in 1893. Entering the Army two years after qualification, he attained his majority in 1906. This gallant officer, who has lately been stationed at Netley, and may well have looked forward confidently towards early promotion to the higher ranks of his department, has thus given up his life for his country at the early age of forty-three years.

"A man whom it has been a privilege to know, and whose memory will linger as a beautiful and tender thing in the minds of all his friends." Thus, writing in the *Guy's Hospital Gazette*, Sir Arthur Conan Doyle eulogises the late Captain Malcolm Leckie, whose death has only lately been confirmed, although occurring on the field of Mons at the end of August. Qualifying L.R.C.P., M.R.C.S. from Guy's Hospital in 1907, he entered the R.A.M.C. the following year, and quickly made many friends, who were attracted by his kindly, generous, and unselfish disposition. Although stricken down so soon, the late Captain Leckie had already covered himself with glory, and had received the Distinguished Service Order. For some time he had been stationed in Egypt, and when sent to the Front was attached to the Northumberland Fusiliers.

Another young Guy's man who has fallen in action—this time a combatant officer—was Second-Lieutenant George Bernard Monk, who entered the institution last year for the purpose of studying both medicine and dental surgery. He was, as a matter of fact, already a D.D.S. of Michigan. When the war broke out, the late Mr. Monk was doing his second year's work in anatomy and physiology, but decided to interrupt his scientific career by offering his services to his country, and was soon given a commission in the Royal Warwickshire Regiment.

The late Captain Charles Paget O'Brien-Butler, R.A.M.C., was a popular sporting Irishman, famous in his native land as an amateur rider. It is said that a few years ago, when he came out head of the amateur riders' list, on more than one occasion he rode every winner of the meeting. Pursuing his medical curriculum at Dublin, the late Captain O'Brien-Butler qualified L.R.C.P.&S. Ire. in 1905, and soon after entered the R.A.M.C. At the beginning of the war he was in Galway, but left almost immediately for the Front.

The late Captain Cecil A. T. Conyngham, R.A.M.C., was also a Dublin man, and took his M.B. there in 1906. As a student he distinguished himself in athletics. Joining the R.A.M.C., he was for some time in India, and in October last was ordered from Bombay to East Africa, where he met his death on active service.

Among Edinburgh men who have lost their lives in the war must be mentioned the late Captain Ernest Mure Glanvill, who took the degrees of M.B., Ch.B. there in 1901. Before entering the R.A.M.C. he had gained much clinical experience in responsible hospital posts, including those of casualty medical officer to the East London Hospital for Children, assistant house surgeon to the Devonshire Hospital, Buxton, and assistant medical officer to the Paddington Infirmary.

The perils of warfare necessarily include exceptional risks of accident, and those such as the late Lieutenant Douglas Wardleworth, R.A.M.C., who lost their lives from some unexpected mishap whilst on active service, may surely be considered to have given their lives for the Empire just as if they had been killed on the field of battle. The late Mr. Wardleworth was an M.B., Ch.B. of the Victoria University, Manchester, and after qualification was house surgeon to the Royal Hospital, Salford, and house physician to the Royal Infirmary, Manchester. Subsequently he settled in practice at Sheringham, and joined the R.A.M.C. as temporary lieutenant in October.

